# Engaging Under‐Represented Adolescents and Young Adults in Cancer Research: A Qualitative Exploration of Lived Experiences and Engagement Strategies

**DOI:** 10.1002/cam4.71086

**Published:** 2025-08-11

**Authors:** Jenny Duong, Iqra Rahamatullah, Tristan Bilash, Caitlin Forbes, Sharon H. J. Hou, Brianna Henry, Perri R. Tutelman, Sheila N. Garland, Jackie L. Bender, Kaitlyn Hanson, Sapna Oberoi, Fiona S. M. Schulte

**Affiliations:** ^1^ Department of Oncology, Division of Psychosocial Oncology, Cumming School of Medicine University of Calgary Calgary Alberta Canada; ^2^ Faculty of Education Simon Fraser University Burnaby British Columbia Canada; ^3^ BC Children's Hospital Research Institute Vancouver British Columbia Canada; ^4^ Department of Psychology Memorial University St. John's Newfoundland Canada; ^5^ Discipline of Oncology Memorial University St. John's Newfoundland Canada; ^6^ Department of Supportive Care Princess Margaret Cancer Centre Toronto Ontario Canada; ^7^ Dalla Lana School of Public Health University of Toronto Toronto Ontario Canada; ^8^ Department of Pediatrics and Child Health, Max Rady College of Medicine University of Manitoba Winnipeg Manitoba Canada; ^9^ Department of Pediatric Hematology‐Oncology, CancerCare Manitoba Winnipeg Manitoba Canada; ^10^ Hematology, Oncology, and Transplant Program Alberta Children's Hospital Calgary Alberta Canada

**Keywords:** adolescent, cancer, engagement, under‐represented, young adult

## Abstract

**Background:**

Adolescents and young adults (AYAs; 15–39 years) diagnosed with cancer face unique challenges during and after treatment, which have implications for improving cancer care. However, AYA cancer research is limited by the under‐representation of those who identify as Indigenous, racialized, 2S/LGBTQIA+, and those living with disabilities. The aim of this project was to explore lived experiences and identify barriers and enablers to engagement, to inform strategies that facilitate the inclusion of under‐represented AYAs in cancer research.

**Methods:**

Using a community‐based partnership research (CBPR) approach, under‐represented AYAs diagnosed with cancer, along with non‐patient advocates who serve this population, were recruited through social media, snowball, and convenience sampling. Semi‐structured interviews were conducted virtually through Zoom and analyzed using reflexive thematic analysis. Analyses were informed by collaborations with a patient partner and member‐checking feedback.

**Results:**

Interviews were conducted with AYAs (*n* = 17) and non‐patient advocates (*n* = 2). Analyses generated three themes: (1) representation leads to empowerment, (2) person‐centered approaches are a prerequisite to building connections, and (3) structural contexts influence the impact of research.

**Discussion:**

Our findings inform inclusive strategies for future studies to ensure the voices of all AYAs with cancer are represented in research. This study also highlights the importance of CBPR and the positive impact of knowledge derived from lived experiences in shaping research processes and outcomes.

## 1 Introduction

Adolescents and young adults (AYAs), who are between the ages of 15–39 years, are a distinct age group uniquely impacted by cancer [1]. In Canada, over 9000 AYAs are diagnosed annually [2], and while the majority survive, their health in survivorship can remain significantly compromised by cancer or treatment‐related morbidities [3]. Research involving AYAs with cancer, during and after treatment, is limited by a lack of representation of AYAs from equity‐denied social backgrounds, including people who identify as Indigenous, racialized^1^, 2S/LGBTQIA+^2^, and/or living with a disability [4, 5, 6]. Social identities can influence cancer trajectories, as experiences of discrimination in healthcare can impact one's ability to cope with cancer [7, 8, 9]. Furthermore, social identities such as race and gender are social determinants of health inequities, contributing to disparate health outcomes between social identity groups [10].

Western countries have longstanding and ongoing histories of systemic colonization, which create processes of social marginalization towards Indigenous Peoples and racialized communities [11, 12, 13]. The colonial structures of Western health systems contribute to barriers in the delivery of high‐quality, culturally responsive healthcare, along with racist mistreatment in health research [11, 12, 13]. Health disparities in the 2S/LGBTQIA+ community are also influenced by structures that privilege cisgender and heterosexual people [14, 15]. This impacts the ability of healthcare providers to address 2S/LGBTQIA+‐specific health issues adequately and rationalizes discriminatory behaviors [14, 16, 17]. Lastly, our social environment disadvantages and excludes disabled^3^ people; architecturally inaccessible health facilities, unaccommodating equipment in medical evaluations, and denial of coverage on health benefits are some barriers people with disabilities systematically face [18, 19]. Due to these challenges, Indigenous, racialized, 2S/LGBTQIA+, and disabled AYAs with cancer tend to have a lower prevalence of screening and enrollment in clinical trials, experience diagnostic and referral delays, and have a higher cancer incidence with poorer prognoses in Western societies [16, 17, 20, 21, 22]. The barriers these populations face also contribute to mistrust towards the health system and researchers, affecting engagement with these populations [21]. These disparities highlight the importance of understanding how to address these inequities in the cancer experience.

It is essential to recognize the intersecting nature of social identities, as within each social identity group, representation tends to privilege advantaged subgroups and oppress members with compounding marginalized identities [23, 24]. Current research does not address calls to consider this compounded marginalization, as the literature has an oversimplified focus on intergroup variation [13, 16, 25]. Adopting an intersectional lens in health equity‐focused research is necessary to disrupt the unidimensional portrayal of identity and power, allowing researchers to address interacting inequities and understand the complexity of power imbalances in our societies. Furthermore, there is limited research that directly incorporates the voices of under‐represented AYAs to understand their unique experiences, needs, and challenges at the intersection of cancer and marginalization [15], and reducing them to siloed categories perpetuates oppression towards those excluded from our knowledge base. Overall, more research is needed that meaningfully includes AYAs with cancer across intersecting social backgrounds. Therefore, this study aimed to explore lived experiences and identify barriers and enablers to engagement, to inform strategies that facilitate the inclusion of under‐represented AYAs in cancer research.

## 2 Materials & Methods

### 2.1 Study Design

A community‐based partnership research (CBPR) approach was adopted to create more equitable partnerships between researchers and the patient community we aimed to serve [26]. The principles include recognizing patient partners as active contributors to the research process, recognizing the legitimacy of their lived experiences as knowledge, and conducting research to meaningfully contribute to their communities' wellbeing. Two patient partners (IR, TB) who identify with an under‐represented social identity and lived experience with cancer were recruited from the principal investigator's network. The patient partners collaborated on all stages of the study from protocol design, recruitment, co‐facilitating pilot focus groups, to analysis and knowledge dissemination, and they were compensated accordingly.

The methodology of this study was informed by a reflexive thematic analysis approach from Braun and Clarke [27]. This study had a phenomenological focus in exploring participants' ideas and experiences and a social constructivist focus on lived experience and how meaning is generated during the process of interpreting data. Due to the nature of reflexive thematic analysis, there was no predetermined sample size [28]. The research team practiced reflexivity by recognizing how one's unique intersection of identities, roles, and lived experiences could influence analyses. Several researchers and patient partners belonged to one or more of the under‐represented social identity groups, including the interviewer (JD), who had intersecting under‐represented identities (see Appendix B).

The study was approved by the Health Research Ethics Board of Alberta—Cancer Committee (HREBA.CC‐22‐0126).

### 2.2 Participants and Recruitment

Eligible AYAs were aged 15–39 years, diagnosed with cancer between the ages 15 and 39, living in Canada, and self‐identified as one or more of the following: Indigenous, racialized, 2S/LGBTQIA+, or living with a disability. Non‐patient advocates who held an academic, clinical, or community organization role in Canada that served under‐represented Canadian AYAs with cancer were also eligible to participate. Participants were English‐speaking and required reliable access to the internet.

Participants were recruited through social media posts, snowball and convenience sampling, and promotions by our community organization partners' (e.g., AYA CAN, Queering Cancer) newsletters and other outreach. Non‐random purposive recruitment strategies were chosen due to their benefits in engaging hard‐to‐reach populations [15]. For non‐patient advocates, existing networks were leveraged, and meetings were initiated with potential community partners who shared research interests. An invitation to participate in this study was provided via email if community partners expressed interest during these meetings.

### 2.3 Procedure

Three pilot focus groups, co‐facilitated by researchers (JD, CF) and patient partners (TB, IR), were conducted to test recruitment materials and scripts. Unanticipated challenges with verifying participants' eligibility arose, and potentially fraudulent participants were identified. A screening protocol and one‐on‐one interviews via videoconferencing were implemented based on patient partner recommendations for the current study.

Participants completed an online eligibility survey and screening call with a researcher. Eligible participants provided written consent and completed a demographics questionnaire, including questions on cancer history for AYA participants and organization details for non‐patient advocates. Semi‐structured interviews were conducted virtually through Zoom by JD; they were up to 60 min in duration and guided by interview scripts with open‐ended questions (see Appendix A). Topics included experience with research (e.g., “Have you participated in research before?”), barriers and enablers (e.g., “What are some things that have prevented or disrupted your engagement in research?”), and broader engagement strategies and considerations (e.g., “How can we keep people engaged in research?”). Participants were compensated with a $50 gift card. The interviews were audio‐recorded, transcribed verbatim, and de‐identified prior to analyses.

### 2.4 Data Analysis

**FIGURE 1:**
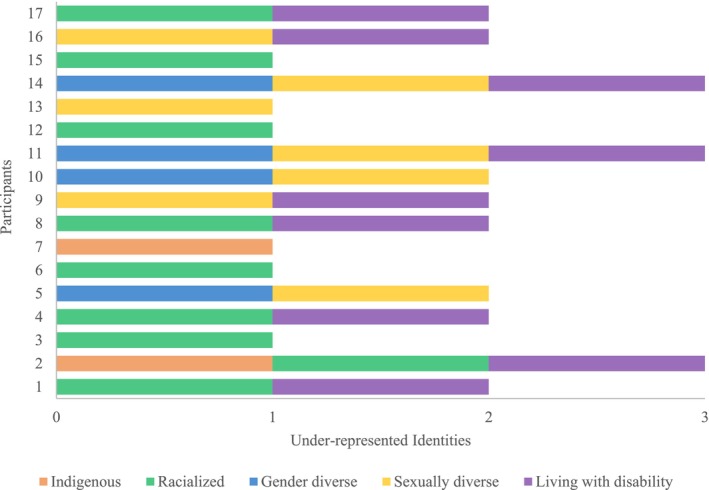
Intersecting under‐represented social identities of AYA participants (*n* = 17). The bar graph visually depicts each AYA participant's unique configuration of under‐represented social identities, including identifying as Indigenous, racialized, gender diverse (e.g., transgender, nonbinary, gender‐fluid), sexually diverse (e.g., gay, lesbian, bisexual), and/or living with a disability.

Demographic data was analyzed with IBM SPSS (version 28) using descriptive statistics. Interviews were analyzed in NVivo (version 14) with an inductive reflexive thematic analysis approach informed by Braun and Clarke [27]. Five members of the research team (JD, FS, IR, BH, SH), including a patient partner, reviewed the transcripts to familiarize themselves with the data. Each transcript was iteratively co‐coded by two members. An initial code list was developed based on findings from the pilot focus groups. Through dialogic critical reflection amongst the research team, the code list was refined, and patterns in the codes were abstracted to prominent themes. Consensus on the themes was established at a final meeting with all reviewers. Lastly, in member‐checking focus groups [29], 12 AYA participants reviewed the visual presentation of under‐represented identities (see Figure 1), along with themes, subthemes, and quotes. Changes were made to emphasize a person‐centred approach as a theme instead of a subtheme.

## 3 Results

### 3.1 Participants

Seventeen AYAs with lived experience of cancer and two non‐patient advocates participated in semi‐structured interviews and were included in analyses (*N =* 19). The mean age of the AYA sample was 29.4 years (SD = 5.3), and the mean age at diagnosis was 23.0 years (SD = 7.9) (see Table 1). With consideration for intersecting identities, two (12%) AYA participants identified as Indigenous; nine (53%) as racialized; four (24%) identified with an under‐represented gender; seven (41%) with an under‐represented sexuality; and nine (53%) were living with a disability. For the non‐patient advocates (*n* = 2), one held academic and community organization roles, and one was a healthcare professional.

**TABLE 1:** Demographic and clinical characteristics of AYA participants (*n* = 16*).

	Mean (SD), range	*n* (%)
Current age (years)	29.4 (5.3), 20–36	
Age at cancer diagnosis (years)	23.0 (7.9), 15–33	
Gender		
Cisgender woman		12 (75.0%)
Nonbinary		4 (25.0%)
Sexuality**		
Asexual		1 (6.3%)
Bisexual		3 (18.8%)
Gay or lesbian		1 (6.3%)
Heterosexual		8 (50.0%)
Pansexual		1 (6.3%)
Queer		4 (25.0%)
Undisclosed		1 (6.3%)
Race/ethnicity**		
Indigenous		2 (12.5%)
First Nations, Metis, or Inuit		1 (6.3%)
Outside Canada		1 (6.3%)
Black/African/Caribbean		3 (18.8%)
Latin American		1 (6.3%)
Middle Eastern		3 (18.8%)
South Asian		2 (12.5%)
Southeast Asian		1 (6.3%)
White/European		9 (56.3%)
Immigration status		
Born in Canada		10 (62.5%)
Immigrant to Canada		6 (37.5%)
Living with a disability		
Yes		9 (56.3%)
Related to cancer and/or treatment		7 (43.8%)
No		7 (43.8%)
Urban		16 (100.0%)
Province		
British Columbia		1 (6.3%)
Alberta		3 (18.8%)
Ontario		9 (56.3%)
Quebec		2 (12.5%)
Newfoundland and Labrador		1 (6.3%)
Religious affiliation		
Christianity		4 (25.0%)
Islam		2 (12.5%)
Sikhism		1 (6.3%)
No religious affiliation		9 (56.3%)
Education		
Some university/college, no degree		3 (18.8%)
University/college, Bachelor's degree		9 (56.3%)
Graduate/Professional school, Master's degree or PhD		4 (25.0%)
Employment		
Working full‐time		5 (31.3%)
Working part‐time		4 (25.0%)
Unemployed		2 (12.5%)
On long‐term disability		2 (12.5%)
On health leave		2 (12.5%)
Other		1 (6.3%)
Income		
Less than $25,000		3 (18.8%)
$25,000–$49,999		6 (37.5%)
$50,000–$74,999		2 (12.5%)
$75,000–$99,999		2 (12.5%)
More than $100,000		2 (12.5%)
Undisclosed		1 (6.3%)
Cancer diagnosis		
Blood		4 (25.0%)
Brain		2 (12.5%)
Breast		1 (6.3%)
Colorectal		2 (12.5%)
Gynecologic		2 (12.5%)
Melanoma		1 (6.3%)
Nasopharyngeal carcinoma		1 (6.3%)
Sarcoma		3 (18.8%)
Cancer care**		
Pediatric		4 (25.0%)
Adult		14 (87.5%)
Cancer treatment status**		
Currently undergoing treatment		4 (25.0%)
Completed treatment		12 (75.0%)
Time since treatment completion (years)	3.2 (4.5), 0.3–15.9	
Recurrence or second cancer diagnosis		4 (25.0%)
Living with metastatic cancer		3 (18.8%)

* One participant requested to have their demographic data removed.

** Equates to greater than 100% as participants could make multiple selections and/or descriptions.

### 3.2 Themes

**FIGURE 2:**
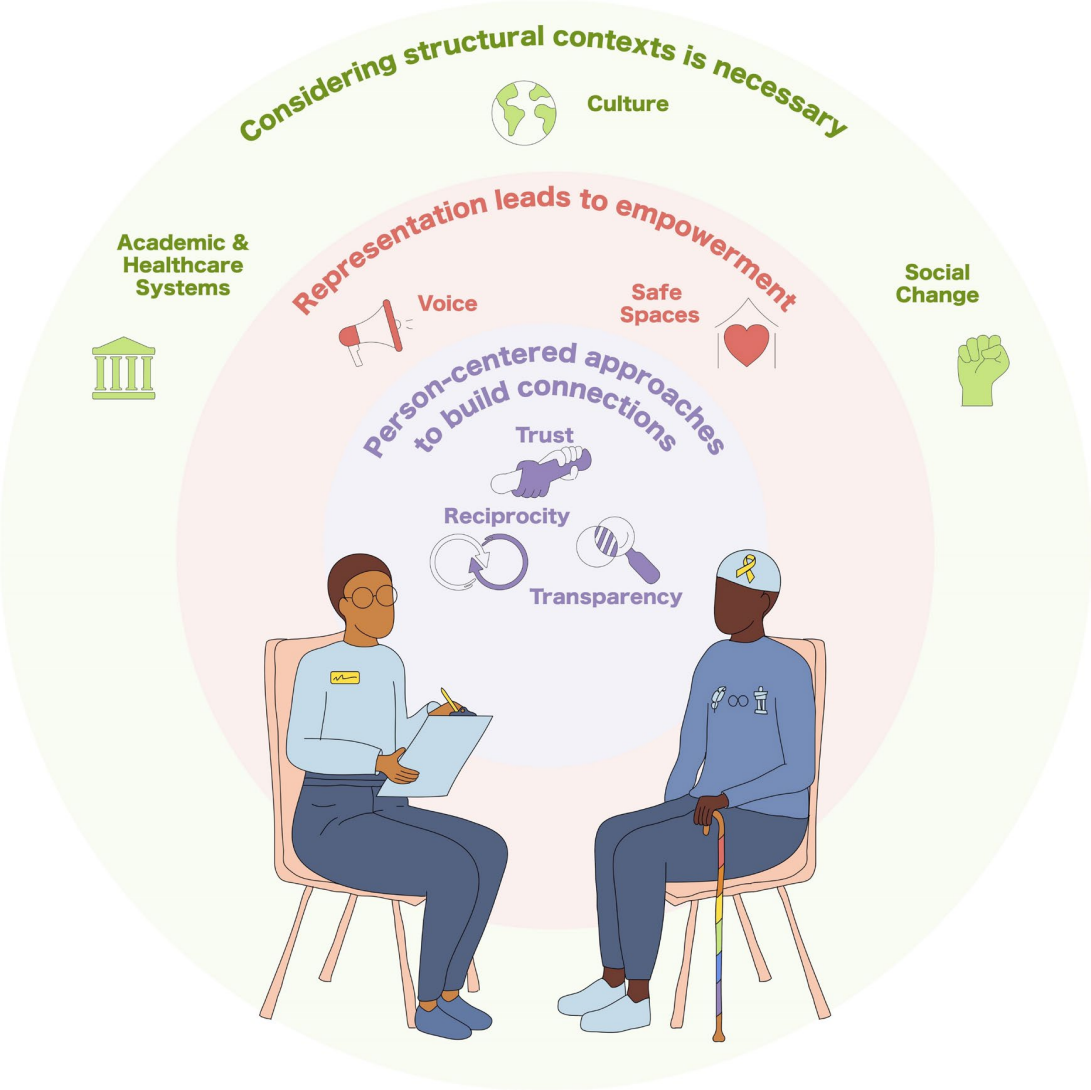
Model summarizing study themes. The model visually depicts interactions between the three themes, presented in an ecological model. The theme of a person‐centred approach to build connections between the researcher (on the left) and participant (on the right) is situated within a context of representation and a broader structural context. Subthemes are included within each theme.

Three themes were generated from the data: “Representation leads to empowerment”, “Person‐centred approaches are a prerequisite to building connections”, and “Structural contexts influence the impact of research”. Within each theme, there were two to three subthemes that highlighted specific considerations in relation to the larger themes (see Figure 2 and Table S1).

#### 3.2.1 Representation Leads to Empowerment

Participants acknowledged how empowering it felt to see other people that shared their own identities in AYA cancer research, healthcare, and advocacy. Representation was recognized as a core facilitator for participating in research, as they felt more likely to be seen and understood when sharing their lived experiences.

Representation matters, and I think people would be more willing to participate in research if they know that members of their community are supporting it and leading it. (AYA, First Nations and White, cisgender woman, heterosexual) ^4^

Almost all participants discussed the importance of representation of social identity, as it was relevant to their experiences in healthcare and research. They valued the intuitive understanding shared by members of their social identity groups. Several participants noted that the identities the study interviewer (JD) shared helped them feel safe and understood.

You had your pronouns in your email and everything, that makes me feel like, ‘OK, those people understand me, they will not think that it's weird that I say my pronouns and that I am a part of the LGBTQ community’. (AYA, lesbian)

Some AYA participants also described compounding experiences of under‐representation. First, AYAs often described entering spaces or receiving resources that lacked representation of people their age. These experiences exacerbated feelings of isolation, as AYAs struggled to find other patients and resources that reflected themselves. Some AYAs also discussed having rarer subtypes of cancer, recognizing some cancers (e.g., breast cancer) had considerable recognition in research, which they felt was missing for their cancer type. These experiences of under‐representation often intersected with participants' social identities.

I haven't found a lot of research for melanoma. And then because melanoma is very rare in Black people, I think that there's even less research on it in Black people and how it can show up. (AYA, Mixed Race Black and White, cisgender woman, heterosexual)

Within the theme of representation, subthemes included having a voice that is heard, along with the role of safe spaces and language.

##### 3.2.1.1 Having a Voice and Being Heard

Participants discussed the importance of having opportunities to speak and feeling that their voice was heard and impactful in research. Several participants recognized how feeling heard had a healing effect, as it alleviated feelings of discouragement resulting from past experiences of repeated advocacy without seeing change.

I've gone through so much—so many things during that treatment that I don't want to have to deal with this again. So I feel that's why it's really important to participate in these research studies so you can actually speak up … that our voices matters. (AYA, South Asian, person with a disability)

These conversations were also recognized as helpful from a non‐patient advocate perspective, as hearing directly from AYAs benefitted their practice.

I encourage open lines of communication. Because at the end of the day, we can never [improve] these programs, that are not about the healthcare provider. These are literally all built to provide resources to the client. (Non‐patient Advocate, Healthcare Professional)

##### 3.2.1.2 Creating Safe Spaces and Language

Participants also emphasized the role of safe environments in fostering representation. Strategies to foster safe spaces included avoiding assumptions about participants' identities and experiences, along with using inclusive language to better capture nuances.

There's safety in being able to disclose who I really am. It would just be really nice to not be an ‘other’ box … also the ability to write in what you are. (AYA, White, nonbinary, queer, disabled)

These recommendations were particularly important with consideration to power differentials between researchers and participants, or healthcare providers and patients. For several gender and sexually diverse participants, this impacted whether they felt safe in disclosing their identities at all.

There is like a fragility or like a vulnerability … I don't want to be seen as making life difficult for them for fear of, not retaliation necessarily, but like, my care is in their hands. (AYA, White, queer + trans, non‐binary, person with a disability)

#### 3.2.2 Person‐Centred Approaches Are a Prerequisite for Building Connections

While recognizing similarities in experiences and enablers of research participation, many participants stressed the simultaneous need to consider individual needs and preferences through a person‐centered approach. For instance, some participants emphasized the role of accessibility in facilitating engagement, such as through virtual research opportunities, which mitigated barriers to participation related to one's health, disability, and socioeconomic situation.

The judgement piece gets eliminated a little bit when it's a virtual [session] … it allows more flexibility for me to have days where I don't feel well and can still participate. (AYA, White, cisgender woman, asexual, person with a disability)

However, other participants described a preference for in‐person engagement, which fostered a stronger sense of community with other under‐represented individuals.

I think would be great in sort of a roundtable thing, where it's an in‐person activity … it would at least make me feel a lot more closer in terms of the engagement. (AYA, Middle Eastern, person with a disability)

Participants supported the presentation of multiple options to participate, along with asking each individual about their needs and preferences, as that demonstrated that they were seen as more than just a number or data point.

Within the theme of developing person‐centered connections, subthemes included building trust, transparency, and reciprocity.

##### 3.2.2.1 Building Trust

Participants highlighted the need to foster trust between researchers and participants to build a meaningful connection and feel confident that the research would benefit their communities. AYAs and non‐patient advocates discussed how beneficial it was to capitalize on pre‐existing trusted connections and spaces. This can include partnering with hospitals or clinicians to recruit patients, using social media platforms like Instagram, and connecting with community groups that represent and serve under‐represented communities to show that the research team is reputable and trustworthy.

My adolescent medicine doctor who I've been seeing for the past three years, she has been on my side relentlessly … when she recommended me for this study I was like ‘I trust you and so therefore I trust that this is a reputable place’. (AYA, racialized, cisgender woman, living with disability)

##### 3.2.2.2 Transparency

Several AYAs highlighted how the present study was clear in its goals to recruit AYAs with cancer of specific identities; this clarity from the start enabled them to make informed decisions to participate.

This particular study being geared towards under‐represented individuals. Like, being explicitly stated that that's who you're looking for. It encouraged me to reach out. (AYA, white, nonbinary, queer)

Many AYAs and non‐patient advocates also discussed continued transparency beyond data collection. Several AYAs were explicit in how hearing study results motivated long‐term engagement, as they felt meaningfully connected with the research team and could see the impacts of the research.

If they're giving me updates and they're letting me know what's going on and things like that, I feel like that would create more of a connection for me and more of an incentive because I'm more involved. (AYA, Middle Eastern, cisgender woman)

##### 3.2.2.3 Reciprocity

Participants discussed the importance of providing compensation to show respect towards participants' contributions to research, and to create a relationship based on the mutual, equitable exchange of benefits between researchers and participants.

A lot of [studies] that have the time commitment of a part‐time job don't have compensation … it almost feels like no respect or payoff personally. Which isn't why I do this, but it's nice to have a little bit of give and take with it and to feel appreciated. (AYA, White, cisgender woman, queer/bisexual, disabled)

#### 3.2.3 Structural Contexts Influence the Impact of Research

Participants discussed the systems that create and perpetuate the marginalization of under‐represented social identity groups in research, healthcare, and everyday life. These contextual factors explained the importance of the aforementioned engagement strategies in addressing gaps in these systems. Subthemes included academic and healthcare systems, cultural contexts, and the desire for social change.

##### 3.2.3.1 Academic and Healthcare Systems

First, participants noted remaining gaps in the literature on the intersection of gender diversity, disability, and the immigrant experience with cancer, which they wanted to see addressed. Furthermore, some participants who were familiar with academic systems recognized how academic cultures promote competition for research funding and publication, when participants felt that research should prioritize having a meaningful societal impact.

You know at the core what we need. … Research is sort of dragging things on because it is providing us, in this economic and capitalist society, with a job. (AYA, racialized)

Some AYAs and non‐patient advocates also described large gaps between existing research and its translation into clinical practice.

It takes approximately 20 years for information to come from research and trickle down into how it actually plays out in provider care behaviour. (Non‐patient Advocate, Researcher and Community Organization Representative)

Participants were aware of how gaps in research and delays in knowledge translation impacted their healthcare experiences, which included discrimination by healthcare providers across clinical environments and a lack of resources specifically for under‐represented AYAs. These experiences demonstrated an overarching culture of exclusion, where participants were burdened with the responsibility to advocate for their own needs, and they wanted to see efforts to address these issues in the systems meant to serve them.

I wished there was like someone [that] came and walked us through support groups or something that existed for various under‐represented groups … Instead of being the individual that had to find them. Because a lot of people just don't have the energy for that when you're super sick. (AYA, queer/bisexual)

##### 3.2.3.2 Culture

Several AYA participants also acknowledged the influence of culture on how others perceive cancer, and the need for cultural responsiveness with AYAs and their families. Some AYAs described how families may immigrate from cultural contexts where systems have untrustworthy practices towards the use of patient data, contributing to distrust towards the Canadian healthcare system and associated health research.

We came from a refugee civil war‐type area where the governments are corrupt. So my parents and I were worried they're going to have all my genetic material and all this stuff stored. (AYA, South and Southeast Asian, Indigenous, person with a disability)

A few participants discussed how cancer can be stigmatized, leading to ostracism and negative impacts on one's relationships. These cultural influences affected whether spaces were considered safe by under‐represented AYAs to disclose their cancer experiences.

There's a lot of survivors who I've met where cancer is completely stigmatized in their cultures, or it's seen as something shameful … your parents, or you, must have done something shameful in your life to bring shame on the family. (AYA, Black, woman, heterosexual, person with a disability)

##### 3.2.3.3 Social Change

Altogether, these systems and contexts reinforced the importance that participants felt with having their voices represented in research. The main motivating factor for participation in the current study was based around a desire to see the real‐world impact of research and positive changes within academic and healthcare systems.

I feel like something that is really important for a patient, it is to feel a part of a movement. It kind of gives us a hope, and this kind of idea that we are contributing to that. (AYA, Black and Latin American, cisgender woman, heterosexual)

The individual‐ and system‐level factors that participants identified were emphasized as potential strategies to affect meaningful societal changes (see Table 2).

**TABLE 2:** Recommendations for improving engagement with under‐represented AYAs with cancer.

Recommendation	Domain	Related theme or subthemes	Examples
Increase diverse representation	Recruitment materials, social media posts, cancer resources	Representation leads to empowerment Transparency	Specify inclusion criteria Include diversity in visuals (e.g., skin colors, genders, sexualities, ages, abilities) Consider using colors that represent cancer diagnoses (e.g., red for blood cancers)
	Research team	Representation leads to empowerment	Include person(s) with lived experience and/or shared identities Include patient and community partners Include pronouns in emails and initial contacts, if comfortable
	Intersectionality in data collection and analyses	Creating safe spaces and language	Provide write‐in options for participants to divulge nuances in their identities and lived experiences, rather than having participants select “other”/“prefer to not disclose”
Practice reciprocity	Compensation	Reciprocity Academic and healthcare systems	Pay each participant at least a minimum hourly wage Provide monetary and non‐monetary forms of compensation, allowing participants to choose what they prefer Avoid offering honorariums as a 1 in X chance to win, to ensure each participant is compensated equitably Provide cancer resources and information to participants, including support groups and opportunities that foster community for under‐represented AYAs with cancer
	Interaction	Having a voice and being heard Person‐centred approaches are a prerequisite for building connections ○ Building trust Structural contexts influence the impact of research	Ask participants about their needs and preferences before making assumptions, have a “beginner's mind” and be open to not knowing the answers Validate lived experiences Acknowledge positionality, power dynamics, and potential biases in shaping participants' sense of safety Offer multiple methods of engagement (e.g., virtual and in‐person, video and phone call) to promote accessibility and meet participants where they're most comfortable Include captions in video calls Provide interview and focus group questions to participants in advance, to allow for time to formulate responses Avoid pushing participants to disclose details beyond what they are comfortable with sharing, as disclosure is inherently vulnerable and could promote further harm and distrust
	Feedback	Having a voice and being heard	Provide opportunities for feedback at the end of the study (e.g., via anonymous survey) to guide improvements in future studies
Promote transparency	Study progress	Person‐centred approaches are a prerequisite for building connections ○ Transparency	Contact participants through their preferred mode of communication to, at a minimum, provide updates post‐data collection (e.g., analyses, manuscript development, knowledge translation efforts). Frequency will vary based on the length of the study and participant preference (e.g., monthly, quarterly, annually)
Engage with AYA cancer spaces	Social media	Building trust	Use social media to connect with AYAs and community groups that serve them
	Healthcare settings	Building trust	Engage with healthcare providers and cancer centres to capitalize on trusted relationships with patients.
	AYA community groups	Building trust	Connect with moderators of AYA community groups (e.g., on Facebook, in community centres)
Prioritize knowledge dissemination practices that have a meaningful impact		Having a voice and being heard Academic and healthcare systems Social change	Collaborate with participants and patient or community partners to co‐create knowledge dissemination and timely implementation plans Look beyond academic publications to identify strategies that meaningful impact AYAs and their communities Move beyond positivism as the default epistemological approach to knowledge production; contribute more qualitative and mixed‐methods research that recognizes human diversity and lived experience Avoid replicating research questions without ensuring parallel efforts towards knowledge translation into clinical care, community practices, and policies Increase funding opportunities that promote equity, diversity, and inclusion; funding towards community partnered research and research products (e.g., educational materials for clinics and community centres) that prioritize community impact Involve participants and patient partners in the direct dissemination of study findings (e.g., as co‐presenters in conference presentations or on social media)

## 4 Discussion

The findings of this study highlighted practical and structural considerations to meaningfully engage with under‐represented AYAs in cancer research. Participants acknowledged gaps in diverse representation in research, which motivated their participation in the current study and informed strategies to address this gap. Conversations about the importance of prioritizing the voices of AYAs also emerged, with participants sharing their experiences of discrimination and exclusion by researchers and healthcare providers. These experiences contributed to their desire to advocate for research that addresses their unmet healthcare needs, in part through promoting systemic changes that support diverse representation in research and tailored clinical care.

Many of our findings align with recent AYA oncology studies, which show that AYAs with cancer generally appreciate a person‐centered approach to their care. For instance, AYAs report a desire for social connection with similar‐aged peers with cancer, along with open communication with their healthcare providers for informed decision‐making [30, 31, 32]. Studies focused on under‐represented groups emphasize person‐centered strategies related to reciprocity, transparency, and trust, like compensating participants and taking a collaborative approach in patient‐provider relationships to achieve shared goals in patients' care [33, 34]. These strategies can be especially beneficial for under‐represented AYAs in addressing the broader context of health inequity and mistrust in health systems that under‐represented AYAs continue to experience.

Beyond these person‐centred engagement strategies, other studies highlighted the importance of addressing broader systems of invisibility by advocating for more researchers and clinicians with intersecting identities who can genuinely understand and respond to the needs of under‐represented communities [6, 15, 33, 34]. This growing body of literature shows the interconnectedness of equitable research and healthcare, as the lack of representative evidence limits providers' abilities to meet the unique needs of under‐represented patients and survivors. Therefore, anti‐discriminatory research approaches and knowledge dissemination practices are necessary beyond the academic context to address issues in healthcare and empower under‐represented AYAs with lived experience of cancer [33, 34]. An anti‐discriminatory practice is especially relevant in the context of a growing anti‐inclusive rhetoric that is realized through funding cuts and legislated restrictions on critical scholarship, education, and programs [35, 36].

The similarities across these studies may reflect how members of different equity‐denied communities hold shared lived experiences despite their diversity, due to overlapping histories of marginalization and oppression in research and healthcare [15]. However, it is important to recognize patterns in experiences while avoiding blanket assumptions and practices that would minimize the inherent diversity of under‐represented AYAs with cancer. Altogether, it is vital to adopt person‐centered approaches that humanize the whole individual, while critically considering the broader systems and structures that create shared patterns of inequity across social groups, as a focus on the micro‐ and macro‐level in parallel provides essential insights.

## 5 Recommendations

Several strategies identified in this study can be adopted by researchers to ensure more representation of equity‐denied AYAs in cancer research. These include increasing diverse representation in media and research teams, asking participants about their unique needs and preferences to tailor engagement, and recruiting through trusted networks and spaces like social media (see Table 2). Improving representation in research would produce findings that can more effectively inform healthcare provider education, clinical practice guidelines, and other modalities that improve the delivery of effective, equitable care for AYAs with cancer. Many recommendations overlap with those from healthcare‐focused studies [6, 34], which is also reflected in our participants' tendency to blend research and healthcare experiences together. Therefore, these recommendations may also hold merit for healthcare providers who want to improve their clinical practice.

Beyond these strategies, researchers and healthcare providers are encouraged to acknowledge and critically consider systemic factors, including institutional and cultural barriers that impact under‐representation. This critical reflection can facilitate efforts to genuinely involve and amplify the voices of patients, to address meaningful research questions and implement actions that support their communities. As noted by one of our participants, considerable efforts to reduce knowledge translation gaps are timely, as AYAs may “age out” of this category before improvements in their care can be realized.

## 6 Limitations

Our recruitment occurred mainly on social media platforms, where many participants were engaged as patient partners and advocates. This may have biased recruitment, and efforts to engage AYAs who are not as engaged on social media are needed. Focus groups and interviews were also conducted online, so future research should explore avenues for accessible participation that do not require reliable internet and sufficient technology. Lastly, our definition of “under‐representation” did not capture other social identity categories, including religion, socioeconomic status, immigrant status, language, and geographic location, though this list is non‐exhaustive. Further inquiry into the inclusion of additional under‐represented groups would strengthen our collective efforts towards inclusive research.

## 7 Conclusion

There is a need to address the exclusion of under‐represented groups of AYAs in cancer research, as this knowledge influences whether cancer care is equitable. The findings of this study provide practical strategies and recommendations for cancer research that is inclusive and person‐centered, and acknowledges the role of contexts and systems that shape patterns of inequity. These recommendations complement the rise in research on the healthcare experiences of under‐represented AYAs with cancer, facilitating dialog and further inquiry into the meaningful inclusion of diverse AYAs with cancer in research and the care it informs.

## Author Contributions

**Jenny Duong:** conceptualization (equal), formal analysis (equal), investigation (equal), methodology (equal), project administration (equal), visualization (equal), writing – original draft (equal), writing – review and editing (equal). **Iqra Rahamatullah:** conceptualization (equal), formal analysis (equal), investigation (equal), methodology (equal), visualization (equal), writing – original draft (equal), writing – review and editing (equal). **Tristan Bilash:** investigation (equal), methodology (equal), writing – review and editing (equal). **Caitlin Forbes:** conceptualization (equal), investigation (equal), methodology (equal), writing – review and editing (equal), writing – review and editing (equal). **Sharon H. J. Hou:** conceptualization (equal), formal analysis (equal), methodology (equal), writing – review and editing (equal). **Brianna Henry:** formal analysis (equal), investigation (equal), methodology (equal), writing – review and editing (equal). **Perri R. Tutelman:** methodology (equal), writing – review and editing (equal). **Sheila N. Garland:** conceptualization (equal), methodology (equal), writing – review and editing (equal). **Jackie L. Bender:** conceptualization (equal), methodology (equal), writing – review and editing (equal). **Kaitlyn Hanson:** methodology (equal), writing – review and editing (equal). **Sapna Oberoi:** methodology (equal), writing – review and editing (equal). **Fiona S. M. Schulte:** conceptualization (equal), formal analysis (equal), funding acquisition (equal), methodology (equal), supervision (equal), visualization (equal), writing – original draft (equal), writing – review and editing (equal).

## Conflicts of Interest

The authors declare no conflicts of interest.

## Supporting information


**Table S1.** Themes, subthemes, and quotes from AYA and non‐patient advocate participants (*N* = 19).

## Data Availability

The data that support the findings of this study are available from the corresponding author upon reasonable request.

## Endnotes

^1^
The term “racialized” is used in this study in recognition of the socially constructed nature of categorization based on perceived physical differences. This includes people who identify as Black, East Asian, Latin American, Middle Eastern, South Asian, and Southeast Asian. In Canada, Indigenous Peoples have a unique constitutionally recognized status and are distinguished separately from “racialized” groups in this study.

^2^
The acronym stands for Two‐Spirit, Lesbian, Gay, Bisexual, Transgender, Queer, Intersex, Asexual, and more, to describe members of gender and sexually diverse communities who do not identify as cisgender and heterosexual.

^3^
This article uses a mix of identity‐first and person‐first language to describe people living with disabilities.

^4^
The identifier following each quote represents the participant's AYA or non‐patient advocate status, along with the participant's chosen description of their identities.

## Appendix A

### AYA Interview Guide

#### Non‐patient Advocate Interview Guide

INTRODUCTIONS

Welcome! Thank you for taking time out of your day to attend this interview. My name is (X) and I am (background), and I will be facilitating our discussion today.

Thank you for agreeing to participate in our research study entitled: “We don't know what we don't know: Understanding the experiences of underrepresented adolescents and young adults with cancer in Canada.”

1. The goal of this study is to hear from adolescents and young adults impacted by cancer about their experiences, so we can understand the barriers and enablers of recruitment for participating in research studies.
2. We also hope these interviews will help in building partnerships with AYAs from underrepresented groups and other key stakeholders who work with them.
3. Eventually, we hope to gather a large group of AYAs across Canada who are impacted by cancer and follow them over a few years. We plan to give them questionnaires over time and track changes in their mental health and quality of life and use this information to better inform clinical practices.

Since cancer does not discriminate, we have an obligation to ensure the voices of all AYAs impacted by cancer are heard regardless of how they identify themselves. Thus, we hope that these interviews will help us to develop safe, equitable, and sensitive recruitment strategies to ensure our research represents all AYAs in Canada.

HOUSEKEEPING

*Privacy*: We value your input, and we want to assure you that your identity will not be disclosed to anyone outside the research team. Please make sure that you are in a private space in your home so that you can feel comfortable speaking openly during our discussion. Finally, when it comes to gathering our data and drawing conclusions, while we are interested in your experiences, we will not share any information that could identify you. Other identifying information, such as your name and contact information, or any medical history, such as names of doctors or hospitals, will also not be included in the final data. Rather, we hope to better understand your experiences overall to inform future recruitment strategies.

*Interview guidelines*: In this interview, I will ask you some questions about your experiences participating in research as an under‐represented and/or under‐researched AYA impacted by cancer. I will not be contributing to the discussion, but feel free to ask me to repeat a question if you need to.

Please keep your camera and microphone on. We want this discussion to take the natural course of a conversation as much as possible, so please avoid using the chat box if possible.

*Recording*: I am going to record this discussion, so please speak clearly. Once we complete the recording, we will be referring only to the audio for research purposes. This means that the audio will not pick up actions, such as nodding. So, please try to voice everything.

INTERVIEW QUESTIONS.

To start, if you are willing, please share with us [the name of your community organization], your position, and the population you serve.

Now, we want to learn more about your experiences working with this population.

1. Enablers and barriers
  - What are enablers to engaging under‐represented or under‐researched individuals in your program? In other words, what are some things that have been helpful for you?
  - What are barriers to participation in your program? In other words, what are some things that have been unhelpful, or have prevented or disrupted engagement in your program?
  - What lessons have you learned?
2. Engagement
  - What are some ways we can engage members of under‐represented and under‐researched minority groups?
    ○ Prompts: Recruitment strategies (social media, community groups, advertisements), recruitment materials (posters, advertisements)
  - How can we keep people engaged in research? Especially since we are hoping to follow participants over many years?
    ○ Prompts: What might help keep you interested in continuing research? Incentives? Updates about results?
3. Partnerships
  - One of our main goals of this study is to develop meaningful partnerships with community organizations such as yours. What are some things that are important to you when developing partnerships with groups such as our research team?
    ○ Prompts: Do you currently have any partnerships like this? What are some “lessons learned”?
  - Meaningful partnerships benefit both parties. What types of things does your organization seek when partnering with a group like ours? Ex. research services, infographics, etc.

Thank you for sharing. We are nearing the end of the interview. Is there anything missing from our discussion that you want to share, or any questions I have not asked that you think are important?

CONCLUSION/WRAP‐UP.

Thank you very much for taking the time to participate in our research study. I am very grateful. The information you shared with me today will be used to ensure our future research represents the voices of *ALL* AYAs in Canada who are impacted by cancer.

We want to appreciate your time today and will be in touch within the next couple of business days to provide your gift card.

Once we have finished gathering this data, our plan will be to bring everyone back together to share the outcomes of this research. We will be looking for different ways to communicate the results of this project to you. We would also be happy to continue to build a relationship with your organizations. If you are interested in discussing further opportunities to work together, let us know and we would be happy to discuss further.

Thank you for sharing your voice and perspectives today!

## Appendix B

### Author Reflexivity Statements

Due to this study's phenomenological and social constructivist focus, the authors recognize the importance of practicing reflexivity in reflecting on how analyses were inherently influenced by their positionality, comprising unique backgrounds, roles, and lived experiences.

JD is a second‐generation Vietnamese immigrant to Canada and identifies as a nonbinary, queer person with a chronic illness, who has experience navigating the healthcare system throughout their adolescence and young adulthood. They completed the pilot phase of this study as part of their honors thesis as an undergraduate psychology student with FS. They acknowledge that their experiences may have shaped biases towards the experiences of participants with shared social identities, making their responses more salient compared to others. They also have reflected on how their healthcare experiences may have influenced interpretations of results related to participants' cancer experiences.

IR and TB are patient partners with lived experience of under‐representation and AYA cancer. IR is a Pakistani‐Muslim, second‐generation Canadian woman who speaks fluent Urdu and English. She has been a cancer patient and survivor for the last 10 years of her life. Her journey has been plagued with challenges—some that she worked through more easily, and others that she struggles with every day of her life. Her interest in physiology and psychology, and her lived experience fuel her through patient advocacy and research work, as well as her ongoing medical training to become a physician (at the time of the publication). She acknowledges that we all have various pieces of our social identities that work on multiple levels and create unique, compounding experiences, and she brings this acknowledgement with her through her advocacy, research, and the care she provides to patients.

TB is a transgender man, oncology social worker, and advanced ovarian cancer survivor. He was diagnosed with Stage 3C ovarian cancer at age 30. TB recognized many years ago that his unique perspective and cancer experience were not meaningfully reflected in the research, healthcare provider training, nor patient support resources at the time. Acutely aware of the importance and irrefutable benefit of cancer patients being able to connect with peers, TB was diligent in his ongoing search for any transgender‐specific cancer patient support organizations and other patients like himself—men (of any age) of trans experience with gynecological cancers—to little avail. From his vantage point working within the cancer care system and recognizing the overall lack of understanding of transgender cancer patients' experiences, TB combined some of his own insights with as much peer‐reviewed information as he could find and began providing presentations to oncology colleagues. From there, TB began receiving invites to share his story at national and international healthcare provider conferences. TB notes that even as a very resourceful and privileged oncology social worker sharing intimate details of his health journey on international stages for several years, it still took him almost 18 years to find other trans people with gynecological cancers. TB is mindful that while there are generational differences between 20 years ago and today, the need for more voices from underserved AYA populations remains.

CF and BH are research coordinators working with FS on researching the psychosocial outcomes of survivors of childhood cancer. CF identifies as a white, cis‐gendered woman, and BH identifies as a queer Métis woman.

SH and PT are postdoctoral fellows and registered psychologists. SH is a first‐generation Taiwanese immigrant to Canada and identifies as a racialized, cis‐gendered woman. SH grew up in a multicultural community, and her experience with cultural diversity over her life strongly influences her research and clinical pursuits. SH is dedicated to diversity and health equity research and championing cultural humility in clinical practice. PT acknowledges her perspectives as an educated cis‐gendered woman who identifies as an invisible minority.

SG is cisgender, white, and identifies as female. She is a clinical psychologist and associate professor in psycho‐oncology and maintains a small private practice where she supports under‐represented young adults impacted by cancer. In this way, her research influences her clinical work, which then informs how she approaches her research. JB is a behavioral scientist and assistant professor in public health, with a research program focused on digital health, cancer survivorship, and implementation science.

KH is a medical student who has also done research on diverse adolescents and young adults with cancer in the Canadian healthcare system. She identifies as a cisgender, white female. She collaborated with the research team through the planning stages as well as during the interview process to work through challenges during the recruitment and screening process. She is honored to be part of this research amplifying minoritized voices and, as a future physician, will incorporate the important lessons learned during this project.

SO is a pediatric oncologist and researcher, with a strong focus on sarcomas, supportive care, and symptom management in AYA oncology patients.

Lastly, FS is cisgender, white, and identifies as female. She is an associate professor and a leader in psychosocial oncology. FS is the principal investigator of this study and oversaw the study process.

Overall, the team reflected on their positionality and used guiding principles and questions for intersectional considerations in the analyses (JD, FS, IR, BH, SH).^1^ The inclusion of patient and community partner perspectives (IR, TB) via the CBPR approach^2^ was also vital in the reflexive process, as their lived experiences and identities facilitated critical reflections of our collective understandings of under‐representation in AYA cancer research.

^1^ Hankivsky O, Grace D, Hunting G, et al. An Intersectionality‐based Policy Analysis Framework: Critical Reflections on a Methodology for Advancing Equity. *International Journal for Equity in Health*. 2014;13(119). doi:10.1186/s12939‐014‐0119‐x.

^2^ Michalak EE, Lane K, Hole R, et al. Towards a Better Future for Canadians with Bipolar Disorder: Principles and Implementation of a Community‐Based Participatory Research Model. *Engaged Scholar Journal: Community‐Engaged Research, Teaching, and Learning*. 2015;1(1). doi:10.15402/esj.v1i1.41.
