# Supplementary material for: Engaging Under‐Represented Adolescents and Young Adults in Cancer Research: A Qualitative Exploration of Lived Experiences and Engagement Strategies
Source: Cancer Med. 2025 Aug 11;14(15):e71086. doi: 10.1002/cam4.71086 (PMC12336665; doi:10.1002/cam4.71086)
Supplement: Supplementary file 1 — Table S1. Themes, subthemes, and quotes from AYA and non‐patient advocate participants (N = 19). [file CAM4-14-e71086-s001.docx]

**Table S1**

*Themes, subthemes, and quotes from AYA and Non-patient Advocate* *participants (*N *= 19)*

| **Themes** | **Subthemes** | **Quotes** |
| --- | --- | --- |
| ***Representation leads to empowerment***  Seeing people who shared one’s identities motivated under-represented AYAs to share their own voices and stories. |  | “Representation matters, and I think people would be more willing to participate in research … if they know that members of their community are supporting it and leading it.” (*AYA, First Nations and White, cisgender woman, heterosexual*)  “You had your pronouns in your email and everything, that makes me feel like, ‘OK, those people understand me, they will not think that it’s weird that I say my pronouns and that I am a part of the LGBTQ community’.” (*AYA, lesbian*)  “If we only looked at gender identity and sexuality, we’d really be missing a lot from people of colour, we’d really be missing a lot from Indigenous folks, we’d be missing a lot from looking at different ages of folks. So I think that that having an intersectional lens is really key in working with young adults… if you’re not taking an intersectional lens you’re not going to engage youth at all. They need to see that in order to trust.” (*Non-patient Advocate, Researcher and Community Organization Representative*)  “I feel there are many nuances and differences between types of cancer. So, for my type of cancer specifically, it is more common in people over 70 years old, and male… So, every time that I am waiting to see my doctor, I feel like I am the only younger person there. And so, I feel like in terms of research, it would be great to always have young people.” (*AYA, Black and Latin American, cisgender woman, heterosexual*)  “I haven’t found a lot of research for melanoma. And then because melanoma is very rare in Black people, I think that there’s even less research on it in Black people and how it can show up.” (*AYA, Mixed Race Black and White, cisgender woman, heterosexual*) |
|  | ***Having a voice and being heard***  Representation included amplifying the voices of people with lived experiences and knowing that they were heard. | “I’ve gone through … so many things during that treatment that I don’t want to have to deal with this again… That’s why it’s really important to participate in these research studies so you can actually speak up … that our voices matters.” (*AYA, South Asian, person with a disability*)  “I’ve never, ever heard of any other research where it’s focused on underrepresented communities, so I'm really looking forward to it. But, it’s just – I think mostly quantitative research, whereas this one actually wants to hear about what I think. Like, a place where I'm not just a number.” (*AYA, Middle Eastern, person with a disability*)  “I encourage open lines of communication. Because at the end of the day, we can never [improve] these programs, that are not about the healthcare provider. These are literally all built to provide resources to the client.” (*Non-patient Advocate, Healthcare Professional*) |
|  | ***Creating safe spaces and language***  Safe spaces and the use of inclusive, nuanced language, foster diverse representation. | “In the breast cancer space, I’ve seen the way that studies are described to say, ‘Oh we’re inclusive’ but they’ll usually still default to the word ‘women’ instead of ‘patients’.” (*AYA, white, nonbinary, queer*)  “A lot of people who are Indigenous are not just Indigenous now; you could have an Afro-Indigenous person. You could have an Indigenous person who is also part of the LGBTQ community. So it’s like I think – again, getting back to like putting people in boxes, I think sometimes we close ourselves off to the idea of just whatever is easiest in research, and I get why they do it but I think it’s not necessarily benefitting anyone.” (*AYA, First Nations and White, cisgender woman, heterosexual*)  “There’s safety in being able to disclose who I really am … it would just be really nice to not be an ‘other’ box.” (*AYA, White, nonbinary, queer, disabled*)  “There is like a fragility or like a vulnerability … I don’t want to be seen as making life difficult for them for fear of, not retaliation necessarily, but like, my care is in their hands.” (*AYA, White, queer+trans, non-binary, person with a disability*) |
| ***Person-centred approaches are a prerequisite for building connections***  Recognizing individual needs and preferences fosters meaningful connections between researchers and participants. |  | “The judgement piece gets eliminated a little bit when it’s a virtual [session] … it allows more flexibility for me to have days where I don’t feel well and can still participate.” (*AYA, White, cisgender woman, asexual, person with a disability*)  “I think would be great in sort of a roundtable thing, where it’s an in-person activity … it would at least make me feel a lot more closer in terms of the engagement.” (*AYA, Middle Eastern, person with a disability*)  “However, I think like if you’re looking at demographics and stuff you still have to look at the fact that like there are certain parts of Canada that still don’t have internet access or there are limitations to that too.” (*AYA, First Nations and White, cisgender woman, heterosexual*)  “Regardless of your identity, if you take a step back and consider the person rather than going through a cookie-cutter approach, then you're going to make all your patients feel seen. Which is important.” (*AYA, white, nonbinary, queer*) |
|  | ***Building trust***  Trust facilitates connection and can be established through connecting with other trusted parties. | “My adolescent medicine doctor who I’ve been seeing for the past three years, she has been on my side relentlessly … So when she recommended me for this study I was like ‘I trust you and so therefore I trust that this is a reputable place’.” (*AYA, racialized, cisgender woman, living with disability*)  “I don’t know if I would’ve had participated if I hadn’t seen somebody else from my community post about it… I was like, “OK, this person can vouch for their experience with the interview process.” There was a trust there.” (*AYA, White, cisgender woman, asexual, person with a disability*)  “It’s literally hearing them, giving them the space they need to build trust for the provider. And just kind of going alongside their journey and giving them the space to enable them to trust you, trust the system. And then in turn it kind of turns into, you know, wanting to learn more about the program, wanting to learn more about my role, and then learning more about the resources that are available.” (*Non-patient Advocate, Healthcare Professional*) |
|  | ***Transparency***  Clear communication for what is expected in a study, along with ongoing communications throughout the study, facilitates long-term engagement. | “This particular study being geared towards under-represented individuals. Like, being explicitly stated that that’s who you’re looking for. It encouraged me to reach out.” (*AYA, white, nonbinary, queer*)  “I can agree to do a study, but if it’s very broad, that can be intimidating because I don’t know what is going to be asked… So I think it would be good to have sort of an agenda or an outline or some questions that can be prepared and sent over via email to understand what the participant is getting themselves into.” (*AYA, racialized*)  “If they’re giving me updates and they're letting me know what’s going on and things like that, I feel like that would create more of a connection for me and more of an incentive because I’m more involved.” (*AYA, Middle Eastern, cisgender woman*) |
|  | ***Reciprocity***  The mutual and equitable exchange of benefits between researchers and participants. | “‘Are there honorariums we can share with the families or patient partners or whoever wants to participate?’ And they say, ‘There are but we’re not allowed to share when recruiting.’ And immediately I won’t share that with my network... they already haven't advocated for us in trying to get us compensation, which means they don’t understand our experience at all.” (*AYA, South and Southeast Asian, Indigenous, person with a disability*)  “A lot of [studies] that have the time commitment of a part-time job don’t have compensation … it almost feels like no respect or payoff personally. Which isn’t why I do this, but it’s nice to have a little bit of give and take with it and to feel appreciated.” (*AYA, White, cisgender woman, queer/bisexual, disabled*)  “From what I understand, the research is, like, to get to understand how we can help those people. But, like, I want to know what you’ve done as well to help the people that you want to have [that] information from… Like, previous research or money that was donated and stuff like that.” (*AYA, lesbian*) |
| ***Structural contexts influence the impact of research***  Systems and contexts that perpetuate the marginalization of under-represented social identity groups in research and other spaces. |  | “They can’t or they don’t want to share any longer in these old systems that are very capitalistic where everyone except me is making- or them are considered of value, that they’re going to be retraumatized.” (*AYA, South and Southeast Asian, Indigenous, person with a disability*)  “The structures and things that we’ve put in place with us in a society; like if we really wanted to see differences and change like it could be done much sooner.” (*AYA, racialized*)  “Micro-aggression is exactly the way I would describe it. And there’s just a bunch of them. And outside of hospital and medical treatment areas you also experience that in your daily life, and so you’re grappling with feeling with it, just in your apartment building. And then you’re also trying to deal with it in a place that you’re meant to feel safe.” (*AYA, racialized, cisgender woman, living with disability*) |
|  | ***Academic and healthcare systems***  Gaps in academic systems reinforce delays in knowledge translation and impacts in healthcare. | “You know at the core what we need. … Research is sort of dragging things on because it is providing us, in this economic and capitalist society, with a job.” (*AYA, racialized*)  “It takes approximately 20 years for information to come from research and trickle down into how it actually plays out in provider care behaviour… the information has to come before care providers are going to change how they practice.” (*Non-patient Advocate, Researcher and Community Organization Representative*)  “It is kind of exhausting when you’re tired from chemo to have someone be like, ‘Well, I just don’t get pronouns.’ So it’s kind of an interesting thing to see there’s a flag-raising happening. And that’s great, but what can we do to train nursing staff so that they’re not badgering their queer patients to better understand what queerness is, you know?” (*AYA, White, queer+trans, non-binary, person with a disability*)  “I wished there was like someone [that] came and walked us through support groups or something that existed for various under-represented groups … Instead of being the individual that had to find them. Because a lot of people just don’t have the energy for that when you’re super sick.” (*AYA, queer/bisexual*) |
|  | ***Culture***  There is a need for sensitivity towards under-represented AYAs’ cultural influences on the cancer experience. | "We came from a refugee civil war-type area where the governments are corrupt. So my parents and I were worried they’re going to have all my genetic material and all this stuff stored.” (*AYA, South and Southeast Asian, Indigenous, person with a disability*)  “There’s a lot of survivors who I’ve met where cancer is completely stigmatized in their cultures, or it’s seen as something shameful … your parents, or you, must have done something shameful in your life to bring shame on the family.” (*AYA, Black, woman, heterosexual, person with a disability*)  “I feel that there is a stigma and a taboo in my culture which I really want to break… It’s not that my bad karma did this to me. I often re-heard this a lot of time. You know, ‘you had done something, that’s why you got this’… especially with people who you love, they say this too.” (*AYA, South Asian, person with a disability*) |
|  | ***Social change***  Engagement in research is motivated by a desire to see societal-level changes. | “I feel like something that is really important for a patient, it is to feel a part of a movement. It kind of gives us a hope, and this kind of idea that we are contributing to that." (*AYA, Black and Latin American, cisgender woman, heterosexual*)  “Just knowing that it’s going to help, that we’re working towards a common goal of making things better.” (*AYA, queer/bisexual*)  “I think for the majority of cancer patients that get into advocacy and research, they're doing it for themselves more than anything, because they want to see a change and they want to see improvement and they want to see the furthering of research. So I feel like anything that proves to them that, is more reason and incentive for them to do it.” (*AYA, Middle Eastern, cisgender woman*) |
